# The role of the rectus sheath block in modern perioperative care for midline laparotomy: a review of the evidence

**DOI:** 10.3389/fanes.2026.1725241

**Published:** 2026-02-13

**Authors:** Marcus Abbawy, David Okoh, Arundhati Binuraj, Finlay Holden, Benjamin Fox, Rajneesh Sachdeva, Jagtar Pooni, Manpreet Singh, Thomas Allen, Saibal Ganguly, Fang Gao-Smith, Tonny Veenith

**Affiliations:** 1 University College London Hospitals NHS Foundation Trust, London, United Kingdom; 2 Walsall Manor Hospital, Walsall, United Kingdom; 3 Department of Anaesthesia, Critical Care and Acute Care Medicine, The Royal Wolverhampton NHS Trust, Wolverhampton, United Kingdom; 4 Dudley Group NHS Foundation Trust, Dudley, United Kingdom; 5 Department of Anaesthesia, The Queen Elizabeth Hospital King’s Lynn NHS Foundation Trust, King’s Lynn, United Kingdom; 6 Department of Anaesthesia, University Hospitals Birmingham NHS Foundation Trust, Birmingham, United Kingdom; 7 Department of Inflammation and Ageing, School of Infection, University of Birmingham, Birmingham, United Kingdom; 8 University of Wolverhampton Faculty of Science and Engineering, Wolverhampton, United Kingdom; 9 JSS AHER- Wolverhampton Centre for Future Health and Policy and Innovation, Mysuru, India

**Keywords:** anaesthesia, laparotomy, pain, periooperative management, rectus sheath analgesia

## Abstract

Perioperative management of pain is crucial in optimising patient outcomes after laparotomy. This review focuses on the rectus sheath block (RSB) and its use in midline laparotomies. This article will examine the current evidence on the clinical efficacy of this method, comparing it to alternative anaesthetic methods and outlining the numerous benefits of its use. The future of the RSB is considered, with an emphasis on where advancements may be achieved and the areas that require further research. We refer to the complications associated with RSB, which are uncommon. If a rectus sheath block is performed in accordance with the evidence-based steps outlined in this review, the likelihood of complications should be minimal.

## Introduction

Postoperative pain following a midline laparotomy is a clinical challenge. It causes patient discomfort and imposes a physiological stress that directly contributes to a cascade of postoperative morbidity. This includes pulmonary impairment, delayed gastrointestinal recovery, and prolonged hospitalisation (1). Inadequately controlled pain is a barrier to achieving critical recovery milestones, such as early mobilisation and effective physiotherapy, increasing the risk of complications like deep venous thrombosis and pneumonia (2). Historically, the management of this severe pain has relied heavily on systemic opioids. While effective, this model is limited by a narrow therapeutic window and a well-documented adverse effects profile. These include respiratory depression, sedation, pruritus, postoperative nausea and vomiting (PONV) and opioid-induced bowel dysfunction, manifesting as postoperative ileus. These side effects actively hinder the recovery process, delaying the return of gut function and early nutrition, prolonging hospital stay (3). The contemporary approach to perioperative care, driven by the principles of Enhanced Recovery After Surgery (ERAS), is a shift away from this opioid driven model. ERAS protocols champion a multimodal, opioid-sparing philosophy integrating multimodal analgesic techniques to maximise pain relief while minimising side effects. The cornerstone is the use of regional anaesthesia to provide targeted blockade of the somatic pain originating from the surgical incision, thereby reducing the systemic opioid requirement and its complications (4).

### Rectus sheath block (RSB)

Within this framework, the rectus sheath block (RSB) has emerged as a particularly valuable tool for pain management. The RSB is a fascial plane block anatomically tailored to provide analgesia for midline abdominal incisions. First described in 1899, the revival is a direct result of a powerful clinical need (5). The clinical “pull” from ERAS programs created a demand for analgesic techniques that can provide pain control without the systemic side effects of opioids or of neuraxial blockade, such as hypotension and motor block. The “technology push”, because of the adoption of ultrasound in regional anaesthesia, transformed the RSB from a blind, less reliable procedure into a safe, reproducible, and effective technique. The RSB thus presented an ideal solution to the clinical challenge defined by ERAS, offering targeted analgesia that aligns with early mobilisation and faster recovery.

### The rectus sheath

**Figure 1:**
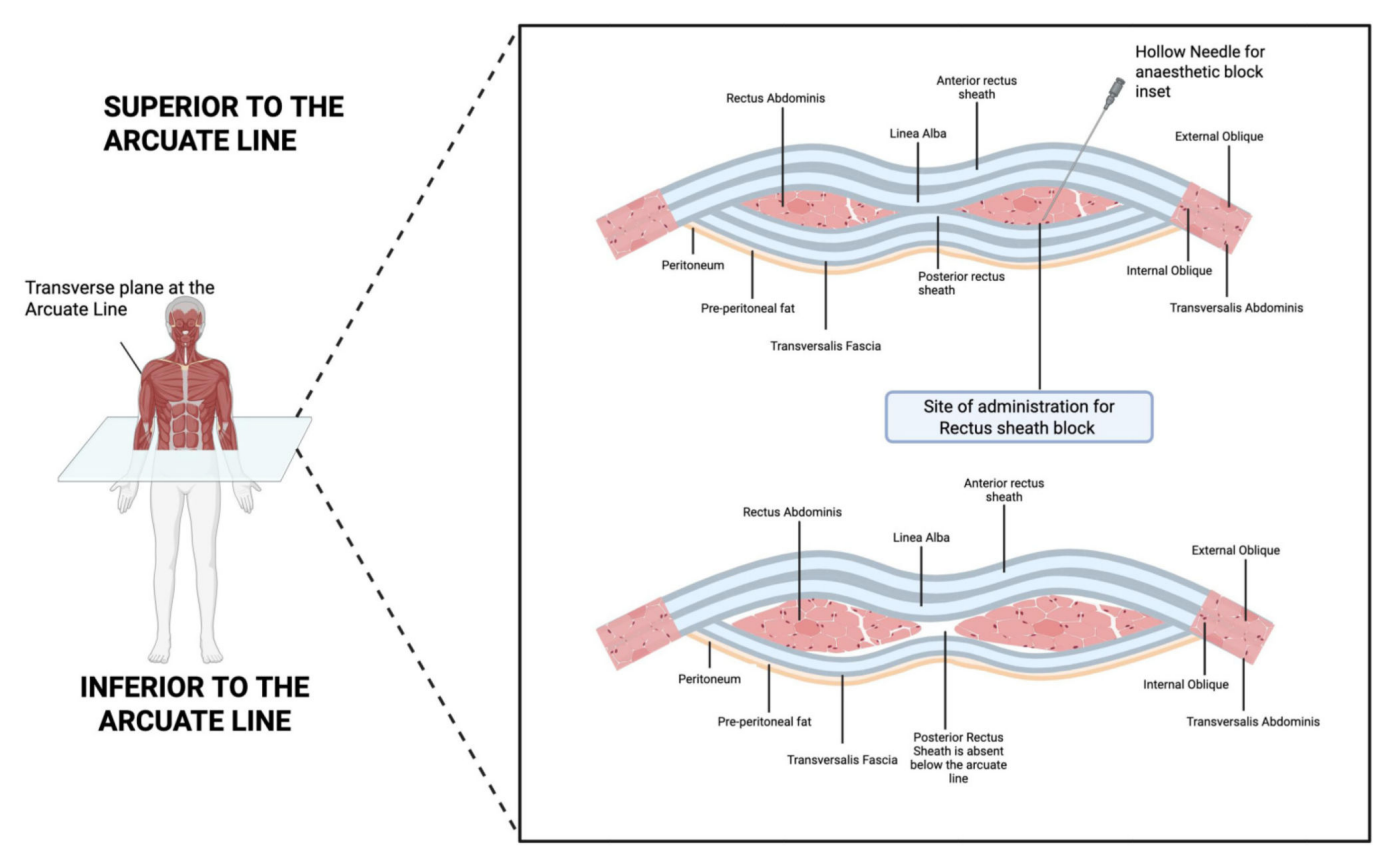
Transverse sections through the anterior abdominal wall: (A) immediately above the umbilicus; (B) below the arcuate line. This cross-section does not illustrate the bilaminar nature of the muscle aponeurosis through which the neurovascular bundles pass. The fibres fuse into a single sheet during the formation of the rectus sheath. The target for insertion of local anaesthetic is the potential space between the posterior surface of the muscle and the hyperechoic posterior rectus sheath. Note that the rectus is supported directly by the transversalis fascia below the arcuate line. (32).

An understanding of the RSB’s efficacy begins with a detailed appreciation of the relevant anatomy (6), as demonstrated in Figure 1. The anterior abdominal wall is defined medially by the paired, vertical rectus abdominis muscles, separated in the midline by the linea alba. Each muscle is enveloped by the rectus sheath, a robust fascial structure formed by the aponeuroses of the three lateral abdominal muscles: the external oblique, internal oblique, and transversus abdominis. A key anatomical feature is the arcuate line, typically located about one-third of the distance from the umbilicus to the pubic crest. Below this line, the posterior layer of the rectus sheath is deficient, leaving only the thin transversalis fascia between the rectus muscle and the peritoneum. This anatomical variation can influence the spread of local anaesthetic in the lower abdomen.

### Neuroanatomy

The innervation of the anteromedial abdominal wall, including the skin and underlying musculature, is provided by the terminal anterior cutaneous branches of the thoracoabdominal intercostal nerves (T7-T11) and the subcostal nerve (T12). After travelling in the neurovascular plane between the internal oblique and transversus abdominis muscles, these nerves pierce the posterior rectus sheath and enter a potential space (6). They travel within this space, between the posterior aspect of the rectus abdominis muscle and the posterior rectus sheath, before finally piercing the muscle and anterior sheath to innervate the skin. This well-defined plane is the target for local anaesthetic in RSB. This unique anatomical arrangement within the sheath makes the block efficient. The rectus abdominis muscle is segmented by three or more tendinous intersections, which are firmly attached to the *anterior* rectus sheath. These intersections do not extend the entire depth of the muscle to fuse with the *posterior* sheath. This creates an uninterrupted, longitudinal fascial plane that allows local anaesthetic deposited at one point to spread freely in both cephalad (superior) and caudad (inferior) directions, thereby blocking multiple dermatomes (T7–T12) from a single injection site or catheter. This anatomical nuance is the reason the RSB provides such predictable and extensive coverage for long midline incisions.

### Vascular structures

The rectus sheath also contains the superior and inferior epigastric arteries and veins, running deep to the rectus abdominis muscle. These vessels are significant, as inadvertent puncture during needle placement is the primary source of complications such as hematoma (7).

### Mechanism of action

The RSB provides somatic analgesia by depositing local anaesthetic (LA) directly into the fascial plane containing the anterior cutaneous branches of the intercostal nerves (8). A bilateral block effectively anaesthetises the anteromedial abdominal wall and periumbilical area, corresponding to spinal dermatomes T9, T10, and T11, which covers the location of a midline laparotomy incision. It is essential to note that the RSB is a somatic block that anaesthetises the abdominal wall (skin, muscle, and parietal peritoneum) and not visceral pain fibres originating from intra-abdominal organs. Therefore, it is most effective as part of a multimodal regimen that includes systemic analgesics for visceral pain.

### Pharmacologic considerations

The efficacy of the block is dependent on the choice of agent, volume, and the potential use of adjuvants. Long-acting local anaesthetics are typically used to maximise the duration of analgesia. Standard options include bupivacaine (e.g., 0.25%) and ropivacaine (e.g., 0.375%–0.5%) (9). Typical volumes range from 10 to 20 mL per side, as the efficacy of any fascial plane block depends on achieving adequate spread of the LA to reach the targeted nerves. A single injection of 20 mL of 0.25% bupivacaine can provide a sensory block lasting approximately 5–7 h.

### Adjuvants for prolongation

To extend the duration of a single-shot block, various adjuvants have been studied. Dexamethasone and the alpha-2 adrenergic agonist dexmedetomidine have been shown to prolong analgesia, potentially through local vasoconstrictive effects, systemic anti-inflammatory actions, or direct effects on nerve fibres, such as inhibiting potassium conductance in nociceptive C-fibres (10). Additionally, adding ketamine to bupivacaine in an RSB resulted in significantly lower postoperative pain scores on movement and reduced 24 h morphine requirements in patients after midline laparotomy (11).

### Best practices for ultrasound-guided RSB

Ultrasound has transformed RSB from a blind, loss-of-resistance technique with a significant risk of vascular injury and inconsistent efficacy (12) to a safe, reliable and reproducible procedure. Ultrasound allows the practitioner to visualise the relevant anatomy, guide the needle to the target plane, and observe the local anaesthetic spread in real-time (13). This has resulted in a higher success rate, shorter onset time, and a significant reduction in complications in clinical practice.

### The ultrasound-guided procedure: a step-by-step approach

**Figure 2:**
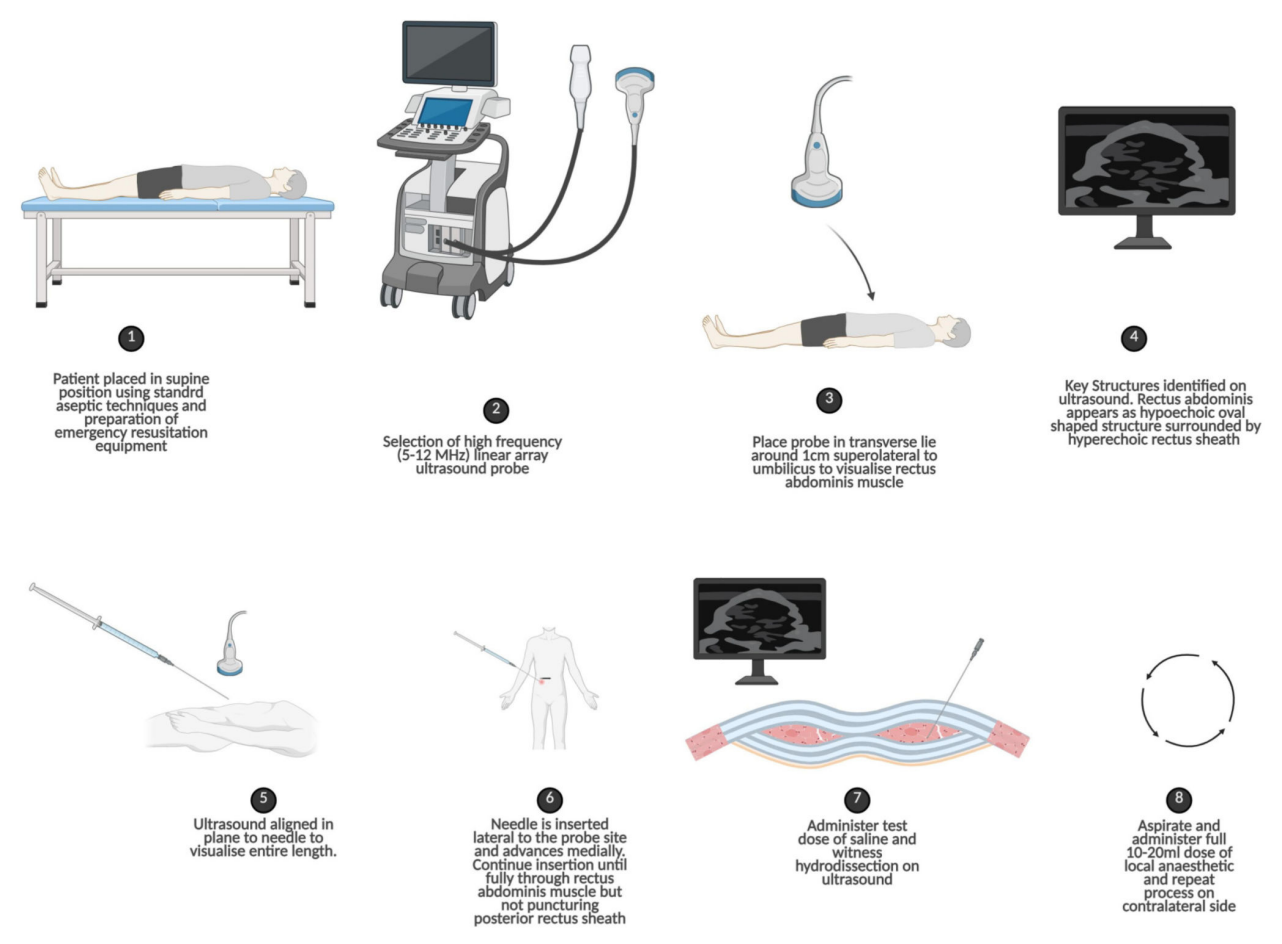
A numbered step-by-step approach to the process of the ultrasound-guided procedure. This process begins with the correct positioning of the patient, followed by the selection of the appropriate equipment and the placement of the probe in the proper anatomical location to identify key structures on the ultrasound scan, such as the rectus abdominis. Steps 5 onwards show the process of aligning and inserting the needle correctly to administer a test dose of saline, followed by the full 10–20 ml dose of local anaesthetic.

Performing a successful and safe ultrasound-guided RSB involves a systematic approach (12), as demonstrated in Figure 2.

#### Preparation

The patient is placed in the supine position. Standard aseptic precautions, including sterile gown, gloves, drapes, and a sterile probe cover, are essential. Emergency equipment to manage potential complications such as local anaesthetic systemic toxicity must be immediately available.

#### Probe selection and placement

A high-frequency (e.g., 5–12 MHz) linear array ultrasound probe is ideal for visualising the relatively superficial structures of the abdominal wall. The probe is placed in a transverse orientation on the abdomen, approximately 1 cm lateral to the midline and just above the level of the umbilicus.

#### Sonoanatomy

The key structures are identified on the ultrasound image. The rectus abdominis muscle appears as a hypoechoic, oval-shaped structure. It is enclosed by the hyperechoic (bright white) lines of the anterior and posterior rectus sheaths (14). Deep to the posterior sheath, the peritoneum is visible as another continuous, hyperechoic line.

#### Needle insertion and targeting

A standard approach is to use an in-plane technique, where the entire length of the needle is visualised. The needle is inserted lateral to the probe and advanced in a medial direction, passing through the subcutaneous tissue and the body of the rectus abdominis muscle. The target is the potential space between the posterior surface of the muscle and the hyperechoic posterior rectus sheath. Tracking of the needle tip is crucial to prevent inadvertent puncture of the posterior sheath and peritoneum.

#### Hydrodissection

Once the needle tip is positioned in the target plane, a small test dose of 0.25–0.5 mL of LA or saline is injected. Correct placement is confirmed by visualising “hydrodissection”—the fluid spreading in the plane and peeling the hypoechoic muscle away from the underlying hyperechoic posterior sheath. After negative aspiration for blood, the full therapeutic volume of 10– 20 mL of LA is injected. The procedure is then repeated on the contralateral side to achieve a bilateral block for a midline incision.

### Single-shot vs. continuous catheter techniques

The choice between a single injection and a continuous catheter technique depends on the anticipated duration and severity of postoperative pain. A single-shot block, lasting 5–12 h, serves as an effective *adjunct* within a broader multimodal plan (15). Its effect will wane, necessitating a transition to other analgesics. In contrast, for major laparotomies where significant pain is expected for several days, a continuous rectus sheath catheter (RSC) is essential. RSC can provide analgesia for 48–72 h via continuous infusion or intermittent boluses, allowing it to serve as a constant postoperative analgesic regimen, much like an epidural (15). This distinction is critical as studies on single-shot blocks may show diminishing effects after 12–24 h. In contrast, those on continuous catheters demonstrate sustained efficacy and are necessary to realise the full potential of the RSB as a viable alternative to neuraxial analgesia.

### Optimal timing: pre-emptive vs. postoperative blockade

Performing the RSB after the induction of general anaesthesia but before the surgical incision is known as pre-emptive analgesia. The theory is that blocking noxious afferent signals before they begin can prevent the establishment of central sensitisation, thereby reducing postoperative pain hypersensitivity and overall analgesic requirements.

A randomised controlled trial in patients undergoing laparoscopic cholecystectomy found that a preoperative RSB resulted in significantly lower total rescue analgesic consumption at 24 h compared to a postoperative block. Preoperative placement also has the practical advantage of allowing for early detection and management of any block-related adverse events before the patient emerges from anaesthesia (16).

#### Postoperative blockade

In some clinical scenarios, postoperative placement is necessary or preferred. If the surgical plan involves the rectus muscle itself, such as the creation of a stoma, the block should be performed after surgery to prevent distortion of the anatomy. The block can also be placed by the surgeon under direct vision during the closure of the abdominal wall or by an anaesthesiologist in the post-anaesthesia care unit as an effective rescue technique for uncontrolled pain.

### Clinical efficacy

#### Postoperative pain scores

The primary goal of the RSB is to reduce the intensity of postoperative pain, both at rest and during movement. The evidence supporting the RSB’s ability to relieve pain at rest is robust. A large and recent systematic review and meta-analysis by Jeffries et al., which included 20 randomised controlled trials (RCTs) with a total of 1,421 participants, provides the most comprehensive data to date (17). This analysis found that patients receiving an RSB had significantly lower pain scores at rest compared to control groups during the immediate postoperative period (0–2 h, *P* < 0.001) and extending to the 10–12 h mark (*P* < 0.001). This is consistent with individual studies that also report lower pain scores in the early postoperative phase. The meta-analysis, however, noted that the pain scores were no longer significant in the 12–24 h period (*P* = 0.11), which likely reflects the waning effect of the single-shot techniques included in many of the trials.

#### Pain on movement/coughing

Effective postoperative analgesia must control static pain and dynamic pain experienced during movement, coughing, or deep breathing (18). This facilitates early mobilisation and prevents postoperative pulmonary complications. Several studies have demonstrated the efficacy of the RSB in this regard. One RCT that utilised a combination of ketamine and bupivacaine for the RSB found a significant reduction in pain scores *on movement* compared to a control group receiving bupivacaine alone (11). By effectively blunting the sharp, incisional pain associated with movement, the RSB plays a crucial role in enabling functional recovery.

#### The opioid-sparing effect

Modern analgesia aims to reduce opioid consumption to limit side effects. The evidence demonstrates a significant opioid-sparing impact of the RSB. The 2024 meta-analysis by Jeffries et al. concluded that 24 h intravenous (IV) morphine equivalent consumption was significantly lower in the RSB group compared to the control group (*P* < 0.001). This conclusion is supported by individual RCTs and case series that consistently report reduced intraoperative and postoperative opioid requirements in patients who receive an RSB.

#### Evidence on opioid sparing

Despite this strong evidence, a degree of uncertainty has been noted in the literature (19). This meta-analysis, which included eight RCTs and 386 patients, found no statistically significant evidence that RSB reduced opioid consumption or pain intensity compared to control groups.

This apparent contradiction is likely not a reflection of the block’s inefficacy but due to methodological differences between the reviews. The Jeffries et al. analysis is more recent, substantially larger (20 RCTs vs. 8), and includes nearly four times as many patients (1,421 vs. 386), giving its conclusions greater statistical power and reliability. The authors of the Jeffries review explicitly stated their goal was to address the “degree of uncertainty” generated by prior, more limited reviews. Abdildin et al. acknowledges that continuous RSB administration demonstrates better results than single-shot techniques (19) (Table 1). It is plausible that by pooling studies of single-shot and continuous blocks without a subgroup analysis, the significant but time-limited effect of single-shot blocks was diluted over the 24 h measurement period, masking the true benefit. Therefore, the positive findings of the larger, more recent, and more comprehensive meta-analysis should be considered the current best evidence.

#### Impact on patient recovery and morbidity

**Figure 3:**
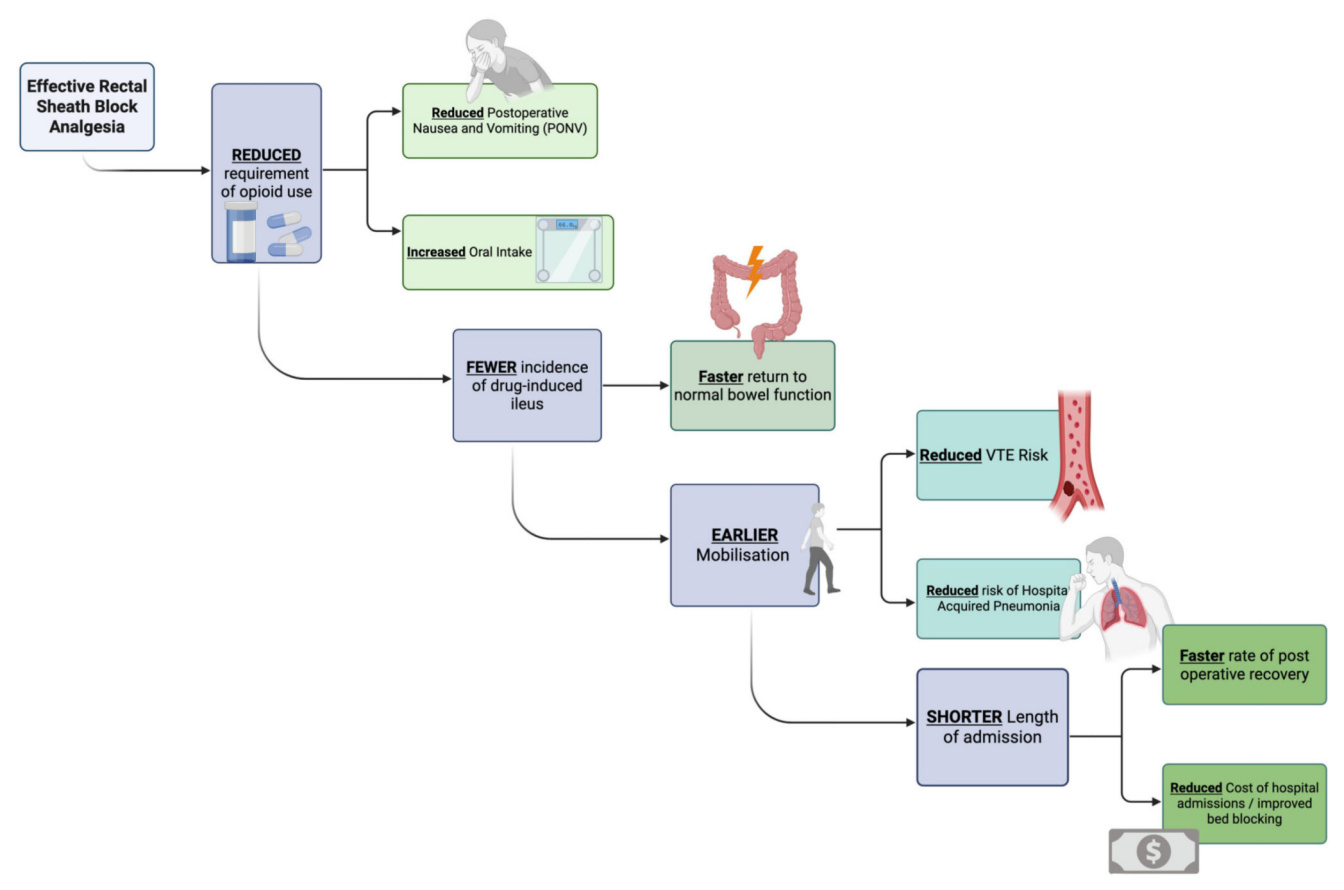
A summary of the downstream benefits of adequate RSB analgesia. These include a reduced postoperative opioid requirement, leading to fewer incidences of drug-induced ileus, earlier mobilisation, and a shorter length of hospital admission. The figure illustrates how this not only enhances postoperative recovery but also has a positive impact on hospitals.

**Figure 4:**
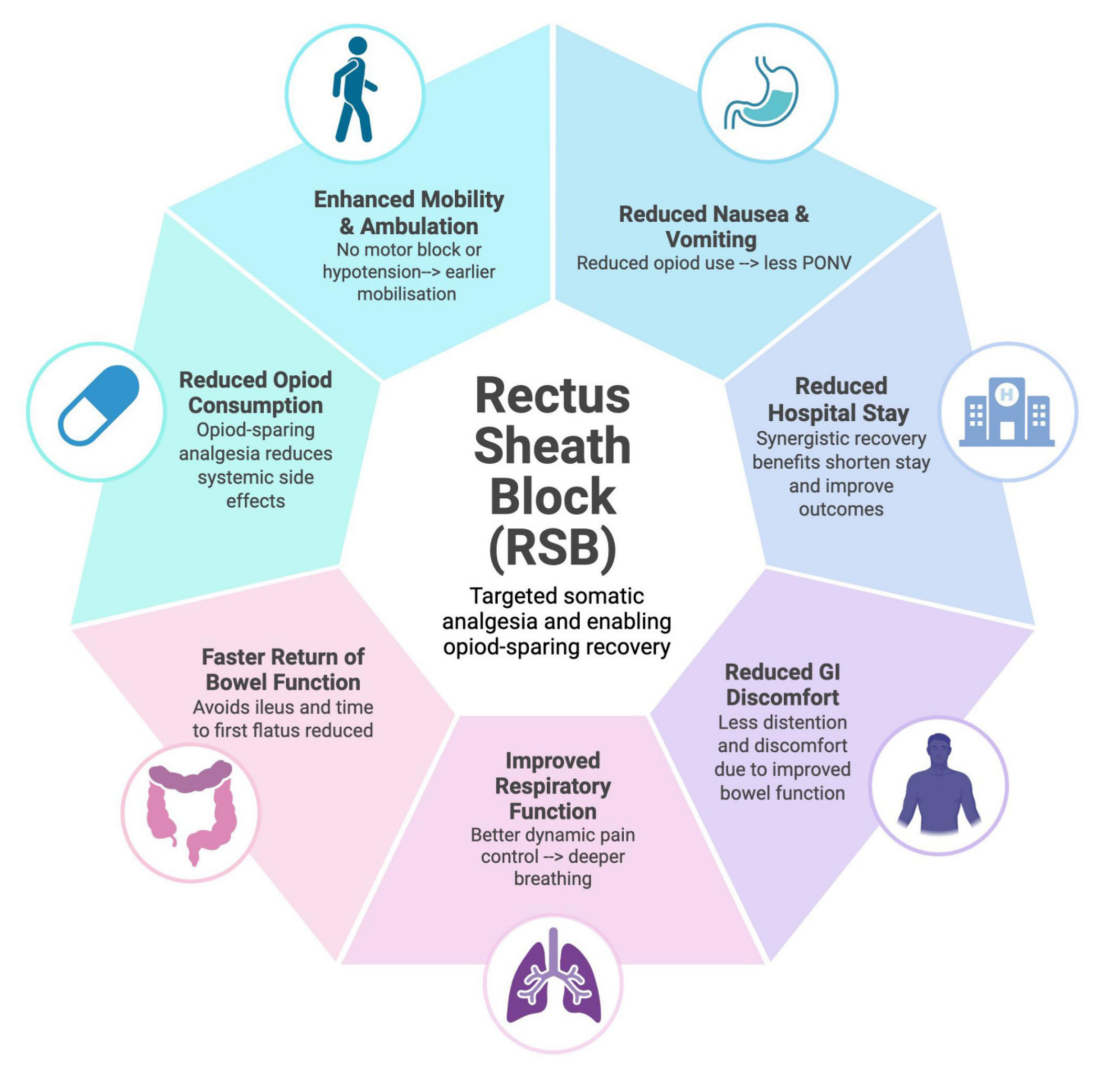
Key benefits of rectus sheath block in perioperative care.

The benefits of the RSB extend far beyond simple pain scores and opioid usage, as demonstrated in Figures 3 and 4. By providing effective, targeted analgesia, the block encourages a positive feedback loop of recovery, where each benefit synergistically reinforces the others, accelerating the patient’s recovery.

#### Enhanced mobility and ambulation

Early mobilisation is the key to ERAS for preventing venous thromboembolism and postoperative pulmonary complications (PPCs). The RSB provides potent somatic analgesia without the side effects of neuraxial techniques, namely, lower limb motor blockade and postural hypotension, which prevent patients from mobilising. This translates directly into earlier ambulation. Comparative studies have consistently shown that patients with RSBs mobilise significantly earlier than those with epidurals. The Tudor et al. study reported a mean time to mobilisation of 2.4 days for the RSC group vs. 3.5 days for the epidural group (*p* < 0.05), while another found a mean time of 7 h vs. 8.28 h (*p* = 0.009) (20). It is a common clinical observation to see patients with rectus sheath catheters walking within 48 h after a major laparotomy.

#### Gastrointestinal benefits

The opioid-sparing effect of the RSB has direct, positive consequences for gastrointestinal function and patient comfort.

#### Reduced postoperative nausea and vomiting (PONV)

PONV is a common side effect of opioid administration that can delay oral intake and recovery. By substantially reducing the need for systemic opioids, the RSB causally minimises the incidence of PONV (21). Multiple studies have reported a significantly lower incidence of nausea and vomiting in patient groups receiving an RSB compared to opioid-based control groups.

#### Faster return of bowel function

Opioid-induced bowel dysfunction is a primary driver of postoperative ileus, a common complication that prolongs hospital stay. The opioid-sparing nature of the RSB helps to avoid this complication, facilitating a rapid return of normal bowel function. One study reported that the time to first flatus was significantly reduced in patients receiving RSB. Another study comparing RSB to thoracic epidural analgesia found that the first bowel movement occurred, on average, two days earlier in the RSB group, highlighting a functional advantage (22).

#### Influence on respiratory function

Severe pain from a midline laparotomy leads to splinting, shallow breathing, and ineffective cough. This results in a restrictive pattern of pulmonary dysfunction and increases the risk of atelectasis and pneumonia. Adequate analgesia through RSB, with control of dynamic pain, can mitigate this respiratory compromise. This allows patients to breathe more deeply and cough more effectively, crucial for clearing secretions and preventing PPCs. While direct, high-level evidence measuring spirometry values, such as Forced Expiratory Volume in 1 s (FEV1) and Forced Vital Capacity (FVC), after RSB for laparotomy is still emerging, a clinical trial is currently underway to investigate this specific outcome.

#### Impact on hospital stay

Synergistic benefits, such as pain control, fewer opioidrelated side effects, earlier mobilisation, faster return of gut function, and potentially fewer pulmonary complications, translate into a shorter duration of hospitalisation. A retrospective study of patients undergoing major gynaecological surgery found a statistically significant reduction in hospital stay for those receiving an RSB, with a median of 4 days compared to 5 days for the control group (*p* < 0.001) (23). The pilot TERSC randomised control trial funded by the UK’s National Institute for Health and Care Research (NIHR) reported a mean decrease in length of hospital stay of 1.8 days in the RSC group, suggesting that the block has the potential for a significant positive health economic impact (24). As was seen with the initial implementation of ERAS protocols, which dropped the average colorectal surgical patients by an average of 3 days (25), there is immediate cost-saving benefits to healthcare systems as well as fewer escalations to intensive care units for additional organ support, further compounding savings (26).

#### Comparative analysis: RSB and thoracic epidural analgesia (TEA)

The clinical utility of the RSB is best understood by comparing it to other established regional anaesthesia techniques for abdominal surgery. The debate between RSB and TEA is shifting from a question of which provides better pain relief to which better facilitates overall recovery. This is demonstrated in Table 2.

#### Analgesic efficacy

For decades, TEA has been considered the “gold standard” for post-laparotomy Pain. However, a growing body of evidence, including a meta-analysis, suggests that continuous RSCs provide **e**quivalent analgesia, with no statistically significant difference in patient-reported pain scores at 24 or 48 h (27). Some studies have even reported consistently lower pain scores in the RSB group (22). While one meta-analysis noted that TEA slightly reduced the requirement for rescue analgesia, the overall pain scores between the two techniques remained equivalent.

#### Side effect profile and functional recovery

The RSB demonstrates a clear advantage in the domains of side effects and functional recovery (28). TEA is associated with a significant risk of sympathetic blockade, leading to hypotension and lower limb motor weakness. These side effects actively hinder key ERAS goals, such as hemodynamic stability and early mobilisation. In contrast, the RSB has a much more favourable side effect profile (18). It provides targeted somatic analgesia with minimal risk of hypotension and no lower limb motor block, directly facilitating earlier ambulation. This makes the RSB a more “ERAS-compliant” technique, as it uncouples potent analgesia from the physiological factors that inhibit recovery.

The superior safety profile of the RSB makes it an invaluable alternative for patients in whom neuraxial techniques are contraindicated. This includes patients with coagulopathy, sepsis, or hemodynamic instability—conditions that are frequently encountered in the emergency laparotomy.

#### RSB and transversus abdominis plane (TAP) block

The RSB and TAP blocks are both fascial plane blocks of the abdominal wall. Still, they are not interchangeable due to key anatomical differences. The TAP block involves injecting LA into the plane between the internal oblique and transvs. abdominis muscles, targeting the thoracolumbar nerves (T6-L1) as they travel through this space, providing analgesia to *the anterolateral* abdominal wall. The RSB, in contrast, targets the terminal anterior cutaneous branches of these nerves after they have entered the rectus sheath, providing dense analgesia to the *midline*.

#### Superiority for midline incisions

Given this anatomical distinction, the RSB is the more logical and effective block for a purely midline laparotomy incision. Evidence from a randomised trial supports this, concluding that a posterior rectus sheath block provided superior analgesia and greater opioid-sparing benefits compared to a TAP block for midline laparotomy. Conversely, for incisions that are more transverse or inferolateral, such as a Pfannenstiel incision for a total abdominal hysterectomy, the TAP block is more effective than the RSB, as the incision falls more within the TAP block’s area of coverage.

#### Complementary and continuous techniques

For surgeries with both midline and lateral components, some evidence suggests that a combination of RSB and TAP blocks can provide comprehensive analgesia. Furthermore, a network meta-analysis of techniques for midline laparotomy concluded that both *continuous* RSB and *continuous* TAP catheter techniques had the highest likelihood of being ranked as the best analgesic options, reinforcing the importance of providing prolonged, sustained blockade for major surgery.

#### Safety profile and risk mitigation

The safety profile of the RSB has transformed with the transition from blind to ultrasound-guided techniques. The discussion has shifted from avoiding the procedure due to unpredictable risks to defining best practices for a visually guided intervention, making it safe for routine clinical use.

#### Potential complications

While generally safe, potential complications include:

- **Vascular Injury and Hematoma:** The primary risk is the puncture of the superior or inferior epigastric arteries, which can lead to a rectus sheath hematoma. This was a concern with blind techniques and underscores the importance of vascular identification in the ultrasound (7).
- **Intraperitoneal/Visceral Injury:** If the needle is advanced deeply, it can breach the posterior rectus sheath and peritoneum, causing bowel puncture or an intraperitoneal injection of LA.
- **Incorrect Injection Plane:** The most common complication noted in a large series was extra-rectus sheath injection, occurring in 2.2% of cases. This includes preperitoneal (0.9%) and intraperitoneal (1.3%) injections, which reduce the effectiveness of the block. Intramuscular injection can also occur if the needle tip placement is too superficial.

#### Quantifying the risk

A large retrospective analysis of 4,033 real-time ultrasound-guided RSBs provides the most comprehensive data available on complication incidence. The study found a low overall complication rate of 2.4%. While rare, serious vascular injuries did occur, with an incidence of 0.2% (29).

Meticulous technique centred on ultrasound guidance is the key to minimising these risks.

- **Pre-procedural Scanning with Colour Doppler:** This allows the operator to visualise and map the course of the epigastric vessels, enabling the planning of a needle trajectory that safely avoids them.
- **Real-Time Needle Tip Visualisation:** Continuous, real-time visualisation of the entire needle, and especially the tip, throughout the procedure. This ensures the needle is advanced to the correct plane and prevents it from passing beyond the posterior sheath into the peritoneal cavity.
- **Confirmation with Hydrodissection:** The use of a small initial test injection of LA or saline to confirm the correct plane via hydrodissection provides a final layer of safety. Visualising the separation of the muscle from the posterior sheath confirms correct placement before administering the full volume.

#### Pharmacologic optimisation

**Figure 5:**
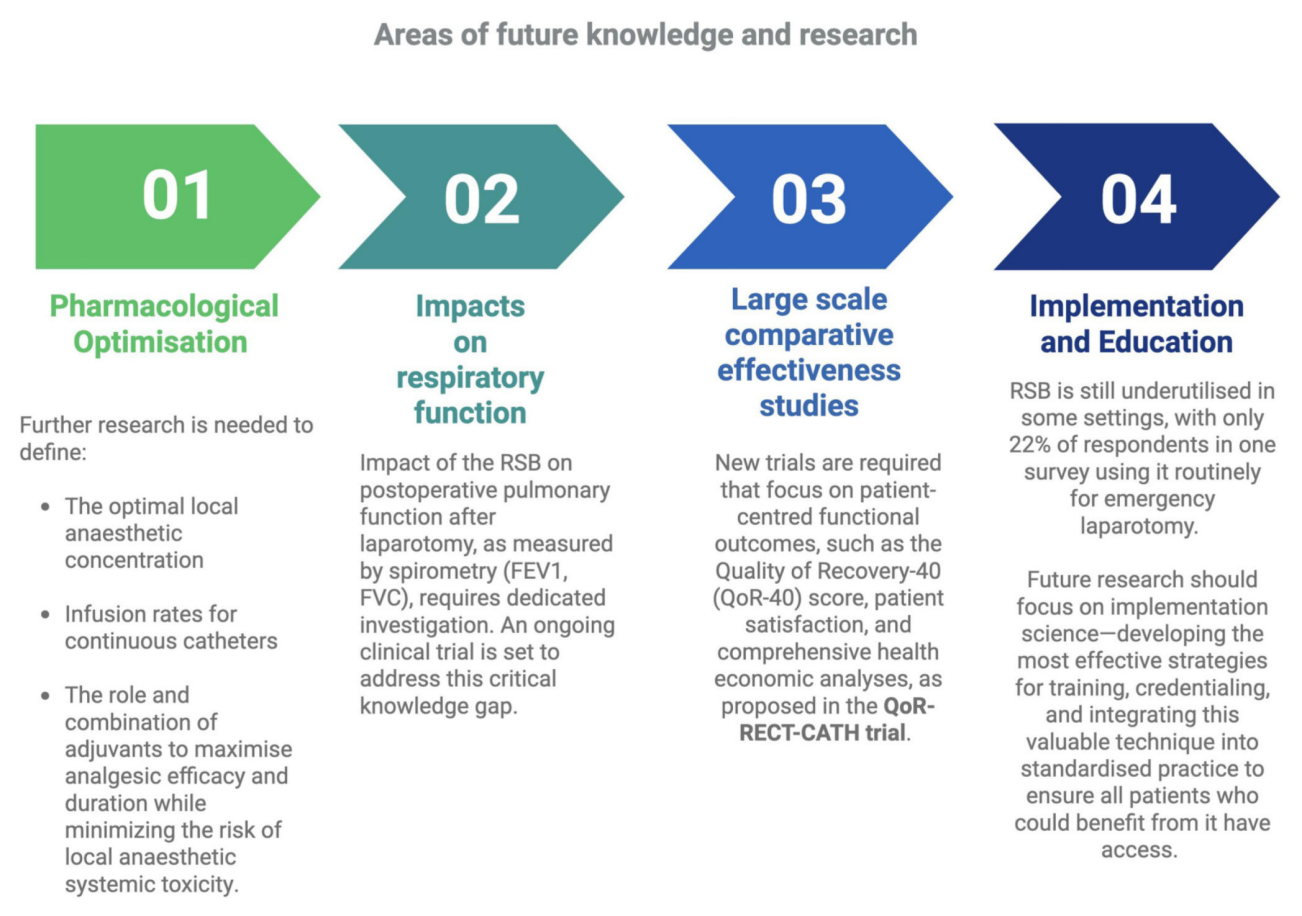
A four step analysis of future knowledge and research.

Further research is needed to define the optimal local anaesthetic concentrations, infusion rates for continuous catheters, and the role and combination of adjuvants to maximise analgesic efficacy and duration while minimising the risk of local anaesthetic systemic toxicity, as demonstrated in Figure 5.

#### Impact on respiratory function

The precise, quantifiable impact of the RSB on postoperative pulmonary function after laparotomy, as measured by spirometry (FEV1, FVC), requires dedicated investigation.

#### Large-Scale comparative effectiveness trials

While existing data are strong, there remains a need for more large-scale, multicentre RCTs that directly compare continuous RSCs with TEA. These trials should focus on patient-centred functional outcomes, such as the Quality of Recovery-40 (QoR-40) score, patient satisfaction, and comprehensive health economic analyses, as proposed in the QoR-RECT-CATH trial (30).

#### Implementation and education

Despite its proven benefits, the RSB is still underutilised in some settings. Future research should focus on implementation science—developing the most effective strategies for training, credentialing, and integrating this valuable technique into standardised practice to ensure all patients who could benefit from it have access (31).

## Conclusion

The ultrasound-guided Rectus Sheath Block, particularly when delivered via continuous catheter, is a safe, effective, and functionally superior analgesic modality for patients undergoing midline laparotomy. The evidence supports its ability to provide potent somatic analgesia, reduce postoperative opioid consumption, and mitigate common opioid-related side effects. These benefits create a cascade of outcomes, including earlier mobilisation, an improved overall recovery, and a reduction in hospital length of stay. The RSB aligns with the core principles of ERAS. It can provide analgesia comparable to the historical gold standard of TEA, but with a markedly reduced sideeffect profile, notably a lack of hypotension and motor block. It is an essential, and often superior, alternative for high-risk patients with contraindications to neuraxial blockade. Despite the strong and growing evidence base, several areas warrant further investigation to optimise the use of the RSB.

## Figures and Tables

**Table 1:** Summary of meta-analyses on RSB efficacy.

Feature	Jeffries et al. (17)	Abdildin et al. (19)
No. of RCTs/patients	20 RCTs/1,421 participants	8 RCTs/386 patients
Search end date	September 2023	October 2021
Surgical scope	Laparoscopic and Open Abdominal Surgery	Abdominal surgery (Adults)
Findings on pain scores	Significantly lower at 0–2 h and 1,012 h. No significant difference at 24 h.	No significant difference in overall pain intensity was observed.
Findings on opioid use	Significantly lower 24 h IV morphine equivalent consumption.	No significant difference in opioid consumption.
Overall conclusion	Rsb is associated with reduced pain and opioid consumption up to 24 h.	No statistically significant evidence in favour of Rsb.

Comparison of findings from two systematic reviews/meta-analyses evaluating the efficacy of Rectus sheath block (RSB) in abdominal surgery. The table summarizes the number of randomized controlled trials (RCTs) and participants included, search end dates, surgical scope, outcomes on pain scores and opioid use, and overall conclusions from Jeffries et al. (17) and Abdildin et al. (19). Jeffries et al. report significant reductions in early postoperative pain and opioid consumption associated with RSB, while Abdildin et al. found no statistically significant benefits.

**Table 2:** Comparative profile of analgesic techniques for midline laparotomy.

Parameter	Rectus sheath block (RSB)	Thoracic epidural analgesia (TEA)	Transversus abdominis plane (TAP) Block
Target nerves	Anterior cutaneous branches of T7–T12	Spinal nerve roots (sensory, sympathetic, motor)	Thoracolumbar nerves (T6-L1) in the fascial plane
Area of analgesia	Anteromedial abdominal wall (midline)	Segmental dermatomes (thoracic, abdominal)	Anterolateral abdominal wall
Efficacy for midline pain	High/Superior	High	Low/Indirect
Opioid-sparing effect	Significant	Significant	Significant (for lateral pain)
Risk of hypotension	Minimal	High	Minimal
Risk of motor block	None (abdominal wall only)	High (lower limbs)	None (abdominal wall only)
Impact on early ambulation	Facilitates	Hinders	Facilitates

Comparison of three regional anaesthetic techniques—Rectus sheath block (RSB), thoracic epidural analgesia (TEA), and transversus abdominis plane (TAP) block—used for postoperative pain management in abdominal surgery. The table outlines their target nerves, areas of analgesia, efficacy for midline pain, opioid-sparing effects, and associated risks such as hypotension and motor block, as well as their impact on early ambulation.
